# Metagenomic Insights Into the Contribution of Phages to Antibiotic Resistance in Water Samples Related to Swine Feedlot Wastewater Treatment

**DOI:** 10.3389/fmicb.2018.02474

**Published:** 2018-10-16

**Authors:** Mianzhi Wang, Wenguang Xiong, Peng Liu, Xiying Xie, Jiaxiong Zeng, Yongxue Sun, Zhenling Zeng

**Affiliations:** ^1^National Risk Assessment Laboratory for Antimicrobial Resistance of Animal Original Bacteria, South China Agricultural University, Guangzhou, China; ^2^Guangdong Provincial Key Laboratory of Veterinary Pharmaceutics Development and Safety Evaluation, College of Veterinary Medicine, South China Agricultural University, Guangzhou, China

**Keywords:** bacteriophages, metagenomics, antimicrobial resistance genes, swine feedlot, wastewater

## Abstract

In this study, we examined the types of antibiotic resistance genes (ARGs) possessed by bacteria and bacteriophages in swine feedlot wastewater before and after treatment using a metagenomics approach. We found that the relative abundance of ARGs in bacterial DNA in all water samples was significantly higher than that in phages DNA (>10.6-fold), and wastewater treatment did not significantly change the relative abundance of bacterial- or phage-associated ARGs. We further detected the distribution and diversity of the different types of ARGs according to the class of antibiotics to which they confer resistance, the tetracycline resistance genes were the most abundant resistance genes and phages were more likely to harbor ATP-binding cassette transporter family and ribosomal protection genes. Moreover, the colistin resistance gene *mcr-1* was also detected in the phage population. When assessing the contribution of phages in spreading different groups of ARGs, β-lactamase resistance genes had a relatively high spreading ability even though the abundance was low. These findings possibly indicated that phages not only could serve as important reservoir of ARG but also carry particular ARGs in swine feedlot wastewater, and this phenomenon is independent of the environment.

## Introduction

The over-reliance and over-use of antibiotics in humans, animals and agriculture has resulted in widespread dissemination of antibiotic resistant bacteria (ARB) and associated antibiotic resistance genes (ARGs). Especially troublesome is the “superbugs” ESKAPE group of pathogens (*Enterococcus faecium*, *Staphylococcus aureus*, *Klebsiella pneumoniae*, *Acinetobacter baumannii*, *Pseudomonas aeruginosa*, and *Enterobacter* spp.) and newly discovered ARGs types *NDM-1* and *mcr-1* (Boucher et al., 2009; Walsh et al., 2011; Liu et al., 2016). Obviously, the emergence and distribution of these pathogens and ARGs highly compromises the clinical treatment and presents a high-risk to public health. Moreover, the ARB and ARGs could also be horizontal transferred by air and water throughout the multiple transmission mechanisms such as transformation, conjugation, and transduction (Andersson and Hughes, 2010; McEachran et al., 2015; Rodriguez-Mozaz et al., 2015; Wang et al., 2015), these processes will further led to an indirect adverse effect to the surroundings ecosystems.

In these transmission processes, the most studied modes are transformation and conjugation, which are mediated by transposons, integrons and plasmids (Harmer et al., 2014; Gillings et al., 2015; Sun et al., 2016). However, the role for bacteriophage (or phage) transduction in HGT has most likely been underestimated. On the other hand, more and more studies have reported that phage genomes originating from animal sources, such as animal farm sludge samples, wastewater samples, soils samples and river water and sediments samples, contain a large reservoir of ARGs (Colomer-Lluch et al., 2014b; Ross and Topp, 2015; Calero-Caceres and Muniesa, 2016; Calero-Caceres et al., 2017; Lekunberri et al., 2017a,b). Therefore, as an important reservoir of ARGs, a thorough insight into phage contribution to antibiotic resistance will be of great importance in animal-associated environments, especially the water samples related to swine feedlot wastewater treatment that may have a significant impact on the surrounding environment.

In this study, we examined treated wastewater and pond water in swine feedlots from farms in Guangdong Province, China. We used whole genome sequencing to determine whether phage genomes carried ARGs and performed a comprehensive analysis of whether ARG-carrying phages contribute to antibiotic resistance.

## Materials and Methods

### Site Selection and Water Collection

**FIGURE 1:**
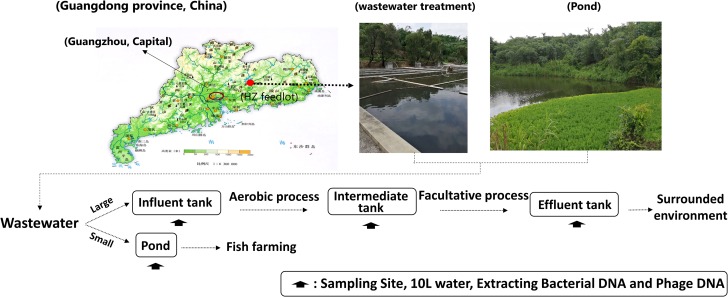
Geographical locations and schematic illustration of water sampling sites.

We examined 16 wastewater samples that included bacterial and phage DNA from the following locations: influent water (INF-B and INF-P), aerobic-facultative intermediate water (AA-B and AA-P), effluent water (EF-B and EF-P), and pond water (PD-B and PD-P). Among these processes, the dissolved oxygen in the aerobic tanks was controlled at 2–4 mg/L and in the facultative tank was controlled at 0.5–1 mg/L (**Figure 1**).

Samples were collected from Huizhou pig feedlots during May 2017. The detailed sampling strategy was adopted from Wang with a few modifications (Wang et al., 2016). In brief: approximately 10 L of surface water were collected; to avoid sampling site fluctuations, water samples were collected four times at 1-h intervals from 9:00 AM to 12:00 AM (a total of 4 h), and then pooled into one composite sample for each respective site (**Figure 1**).

### Bacterial Acquisition and DNA Extraction

Water samples (10 L) were obtained and divided into two aliquots under aseptic conditions. One aliquot was used for DNA extraction from bacteria and bacteriophage and the other was stored at -80°C.

From the 5 L water, 0.5 L was used for bacterial DNA extraction. One hundred and fifty milliliters water was filtered through a 0.22-μm membrane filter (GTTP04700, Millipore, United States) using a vacuum filtration apparatus. The membrane filter was then used to extract DNA using an EZNA water DNA kit (Omega Bio-Tek, United States) according to the manufacturer’s instructions. Then, DNA concentrations were determined using a NanoDrop ND-1000 spectrophotometer (NanoDrop, United States).

### Bacteriophage Acquisition and DNA Extraction

The remaining 4.5 L of each wastewater sample was used for phage DNA extraction. Because of phages could pass through the 0.22-μm filtrate while bacteria are trapped on the membrane surface, we centrifuged the original wastewater of 1.5 L at 3,000 *g* for 15 min to remove solid matter and retained the supernatant. Then, the supernatant was filtered through a sequential series of membranes with pore sizes of 5, 1.2, and 0.45 μm (Midwest Great Technology, Beijing, China) and a final filtration through a 0.22 μm membrane (Millex-GP, Millipore).

After that, all the filtrate was concentrated using a 100 kDa Amicon Ultra centrifugal filter units (Millipore, United States) to a final volume of about 2 mL following the instructions provided by the manufacturer (**Supplementary Figure S1**). Phage DNA extraction was performed according to a published method (Thurber et al., 2009). Briefly, 100 U of DNase I was added to each milliliter of sample and the samples were incubated for 2 h at 37°C. Then, 0.1 volume of 2 M Tris–HCl (pH 8.5)/0.2 M EDTA, 0.01 volume of 0.5 M EDTA (pH 8.0), 1 volume of formamide and 2 volume of 100% ethanol were added to each sample, followed by a centrifugation for 20 min at 8,000 *g* at 4°C to pellet virions. The pellets were then treated with 3 μL of 20 mg/mL proteinase K. Finally, CTAB/NaCl solution, phenol/chloroform/isoamyl alcohol (25:24:1), and 0.7 volumes of isopropanol were used to acquire phages DNA precipitates. The DNA precipitates were then re-suspended in 30 μL of TE (10 mM Tris–HCl, pH 7.5, 1 mM EDTA) for use.

The absence of non-packaged DNA was verified using controls as previously described (Colomer-Lluch et al., 2014a). In brief, an aliquot of each sample was used for the conventional PCR detection of two pairs of different eubacterial 16S rDNA after DNase treatment but before desencapsidation. Only negative samples were used for the subsequent sequencing analysis and others were discarded. The eubacterial 16S rDNA primers were listed in **Supplementary Table S1**. DNA concentrations were measured by UV spectroscopy with a ND-1000 spectrophotometer (NanoDrop, Wilmington, DE, United States).

### Metagenome Sequencing of Water Samples

Bacterial and phage DNA were sequenced using an Illumina HiSeq^TM^ X Ten platform (Illumina Inc., San Diego, CA, United States). Approximately 40 million Illumina sequencing reads were generated (**Table 1**). 16S rRNA gene sequences in each phage DNA fraction were determined using METAXA2 to assess potential bacterial DNA contamination (Bengtsson-Palme et al., 2015). The Comprehensive Antibiotic Resistance Database (CARD) was used for ARG annotation and for ARG grouping according to resistance mechanism (Jia et al., 2017). ARG relative abundance was calculated in relation to the total number of reads in each metagenome.

**Table 1:** Summary of data generated from the metagenomes.

Samples	Influent		Anaerobic-aerobic		Effluent		Pond	
	BAC^β^	PHA^δ^	BAC	PHA	BAC	PHA	BAC	PHA
No. of raw reads(10^6^)	36.1 ± 0.64	39.0 ± 1.86	38.8 ± 0.84	34.7 ± 1.82	33.6 ± 4.48	36.1 ± 5.71	35.9 ± 1.24	34.8 ± 3.24
No. of 16s rRNA reads(10^4^)	NA^γ^	0.18 ± 0.04	NA	0.16 ± 0.03	NA	0.174 ± 0.012	NA	0.095 ± 0.012
16s rRNA (%)	NA	0.0506	NA	0.0459	NA	0.0483	NA	0.0274
No. of ARG^α^ reads(10^4^)	7.7 ± 0.24	0.78 ± 0.24	8.7 ± 1.27	0.23 ± 0.24	7.7 ± 0.24	0.77 ± 0.24	7.7 ± 0.64	0.026 ± 0.0024
ARG reads (%)	0.213	0.0201	0.224	0.007	0.229	0.0213	0.056	<0.001

*^α^ARGs, antibiotic resistance genes; ^β^BAC, bacterial DNA; ^δ^PHA, phage DNA; ^γ^NA, not annotated.*

### Statistical Analysis

Usually, phage transduction depends upon possession of a cognate receptor from the target. If an ARG can be transferred *via* phage in the environment, the communication frequency between phage and bacteria is an indirect measure of the phage contribution to antibiotic resistance and serves as an assessment of antibiotic resistance’s spreading ability (Wang et al., 2018). In this study, the communication frequency was defined as a “spreading ability” value of a phage to spread an ARG by transduction (Eq. 1).

(1) $$\text{Ratio value}=\frac{\text{Relative abundance of ARGs in phage (PH)}}{\text{Relative abundance of ARGs in bacteria (BAC)}}$$

All statistical tests were analyzed in this study were performed with SPSS software (Version 20.0, SPSS, IBM, United States). One-way ANOVA was based on a 5% significance level. A two-tailed Pearson’s bivariate correlation analysis was used to compare bacterial and phage DNA ARG abundance for each antimicrobial. The raw data are uploaded to the database of MG-RAST^1^ and the accession number and detailed information were listed in the **Supplementary Table S2** or access website^2^.

## Results and Discussion

### Abundance of Antibiotic Resistance Genes in Water Samples

According to previous studies, ARG abundance in phages has most likely been overestimated due to the bacterial DNA contamination (Roux et al., 2013; Lekunberri et al., 2017a). Therefore, we screened all unassembled reads from each of the phage DNA samples for 16S rRNA gene. The results showed that all selected influent water, aerobic-facultative intermediate water, effluent water and pond water viromes had low levels of bacterial DNA contamination (i.e., <0.02%) (**Table 1**). These results indicate that downstream analysis of ARG abundance could be further conducted; a relative comparison of ARG types and abundance would reflect phage ARG types. Also, after an annotation to CARD, the relative abundance of ARGs in four kinds of water samples showed some differences.

**FIGURE 2:**
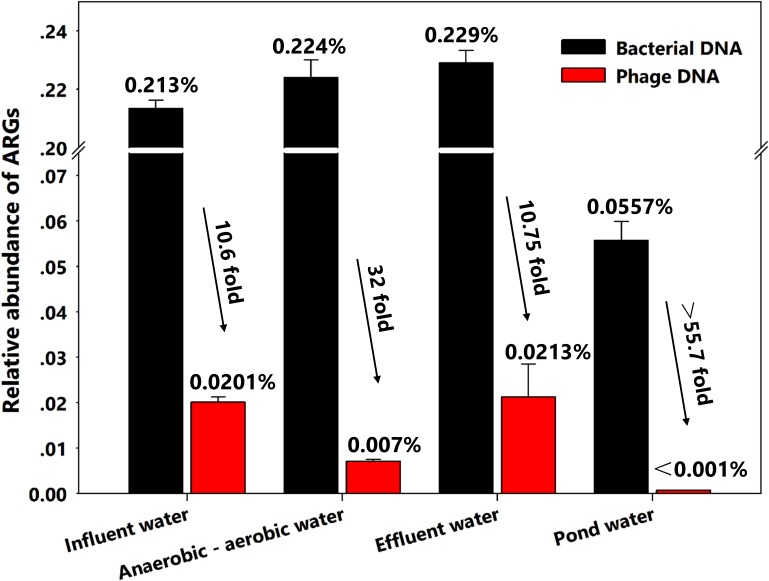
Relative abundance of all antibiotic resistance genes (ARGs) in bacterial and phage DNA from different water samples.

In the wastewater treatment system, the relative abundance of bacterial ARGs in all water samples was always 10- to 33.4-fold greater than that of phages. Surprisingly, though we found a significant decrease in phage ARG abundance between influent and intermediate water samples from 0.0201 to 0.00712%, the ARG elimination effect was not observed when the influent and effluent water was compared and stayed relatively constant at 0.213 and 0.229% for bacterial DNA and 0.0201–0.0203% for phage ARGs, respectively (**Figure 2** and **Table 1**). Moreover, as an adjunct wastewater treatment system, the relative phage ARG abundance (0.001%) in the pond water was 55.7-fold lower than in the bacterial DNA fractions (0.0557%) (**Figure 2**). These results indicate that the swine feedlot wastewater treatment system did not significantly decrease the abundance of ARGs. Meanwhile, phages probably were an important ARG reservoir and play a major role in the spread of antibiotic resistance (Colomer-Lluch et al., 2014a; Subirats et al., 2016; Lekunberri et al., 2017a).

On the other hand, a possible explanation for the higher increase (55.7-fold) in pond water probably is that the low diversity both in bacterial and viral communities may have led to the co-selection of certain bacteria and phages. Yang et al. (2017) have found that ratios of bacteria to virus-like particles in municipal wastewater treatment systems were increased up to 17.5-fold even with low diversity of these communities. This might suggest a co-selection for certain phage types plays a dominant role in the spread of antibiotic resistance. Moreover, phage numbers are estimated to be >10-fold greater than their bacterial hosts in the environment (Pawluk, 2017). The imbalance in the bacteria-phage ratio in wastewater samples might lead to greater numbers of ARGs in phages compared with pond water samples.

However, more experimental and directed data are still needed to support our hypothesis. In addition, the lack of knowledge of the classification and identification of the phages from viral genomes is still a roadblock and it requires more in-depth studies.

### Distribution and Diversity of Antibiotic Resistance Genes in Water Samples

**FIGURE 3:**
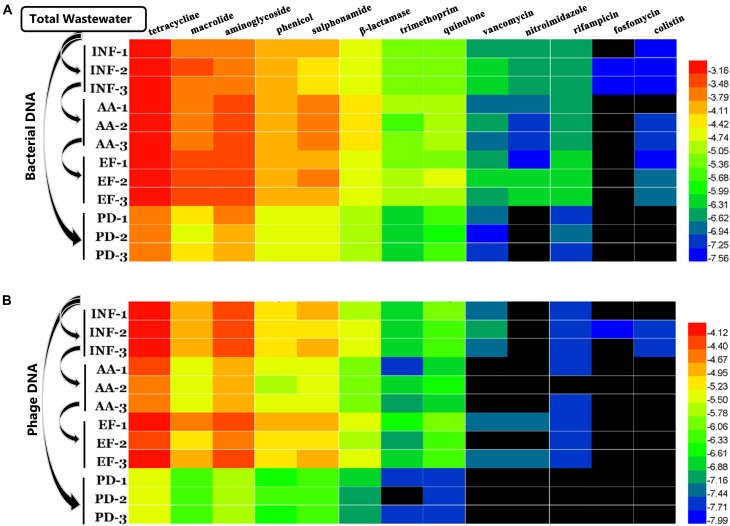
Variations of relative abundance of various ARG types at different locations. **(A)** Bacterial DNA and **(B)** phage DNA. The data were log10 transformed. The black bars indicate that the genes were not detected.

**FIGURE 4:**
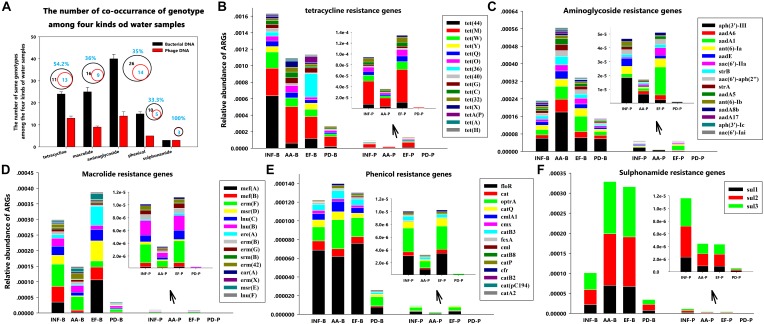
Distribution patterns of ARG types at different sampling locations. **(A)** The number of co-occurrence of genotypes for each antibiotic group among the four kinds of water samples; **(B)** Tetracycline resistance genes; **(C)** Aminoglycoside resistance genes; **(D)** Macrolide resistance genes; **(E)** Phenicol resistance genes; and **(F)** Sulphonamide resistance gene.

Further analysis of the distribution and diversity for different types of ARGs according to the class of antibiotics that they confer resistance to in these two genetic pools, bacteria and virus DNA is needed. Results showed that a total of 13 different types of ARGs between bacterial and phage DNA were detected and tended to a fixed hierarchy of tetracycline ∼ aminoglycoside ∼ macrolide>phenicol ∼ sulphonamide > β-lactams ∼ trimethoprim ∼ quinolone > vancomycin ∼ nitroimidazole ∼ rifampicin > fosfomycin ∼ colistin (**Figures 3**, **4A**). These results were similar to findings from another study using raw sewage (Lekunberri et al., 2017a). However, we were surprised that colistin resistance gene *mcr-1* was present in influent water. Colistin and tigecycline are among last resort of antimicrobials in the treatment of infections caused by carbapenemase-producing *Enterobacteriaceae* (Pitout et al., 2015). The emergence of *mcr-1* might imply that apart from the plasmid-mediated horizontal genes transfer, phage transduction is probably another driving force for HGT in the environment.

Moreover, since we found the same trend for ARG distribution in bacterial and phage DNA fractions, we conducted a Pearson correlation analysis between these two samples based on ARG co-occurrence. All ARG types had significant correlations (*p* < 0.05) except for macrolide resistance genes in effluent water (**Table 2**). These results not only directly demonstrate an intimate correlation between phages and bacteria but also suggest a prediction that the higher the abundance of ARGs in bacteria is, the more the ARGs in phage are. This also implies a greater threat to human health due to greater persistence characteristics of phages (Calero-Caceres and Muniesa, 2016). Thus, for example, *mcr-1*, though we now detected phage-associated *mcr-1* in influent water at a relatively low abundance, this may spread and become problematic for therapeutic treatments in long run.

**Table 2:** Pearson correlations between ARG types in bacterial and phage DNA for each antibiotic class at four water-sampling sites.

Samples	Bacterial DNA/phage DNA			
	Influent water	Aerobic-anaerobic	Effluent water	Pond water
Tetracycline	0.533^∗∗^(*n* = 66^α^)	0.976^∗∗^(*n* = 66)	0.483^∗∗^(*n* = 78)	0.977^∗∗^(*n* = 39)
Macrolide	0.616^∗∗^(*n* = 75)	0.905^∗∗^(*n* = 60)	0.073 (*n* = 72)	0.723^∗∗^(*n* = 24)
Aminoglycoside	0.893^∗∗^(*n* = 96)	0.978^∗∗^(*n* = 84)	0.809^∗∗^(*n* = 99)	0.949^∗∗^(*n* = 42)
Phenicol	0.686^∗∗^(*n* = 36)	0.868^∗∗^(*n* = 36)	0.724^∗∗^(*n* = 36)	0.822^∗∗^(*n* = 15)
Sulphonamide	0.715^∗^(*n* = 9)	0.738^∗^(*n* = 9)	0.188(*n* = 9)	0.636(*n* = 9)
β-lactamase	0.515^∗^(*n* = 39)	0.822^∗^(*n* = 33)	0.649^∗∗^(*n* = 39)	NC^β^(*n* = 3)
Others	NC	NC	NC	NC

*^α^N, The number of ARGs that were co-detected both in bacterial DNA and phage DNA, ^β^NC, not calculated because of the absence of ARGs co-occurrence, ^∗^P < 0.05, ^∗∗^P < 0.01.*

Furthermore, during careful screening of independent genotypes for each antibiotic group, we abandoned the downstream genotype analysis for vancomycin, nitroimidazole, rifampicin, fosfomycin and colistin groups due to absence of ARGs in phage DNA. We found that, though tetracycline resistance genes had the highest abundance (**Figure 3**), the highest ARG diversity was the aminoglycoside group (40 different genotypes in bacterial DNA and 14 different genotypes in phage DNA among four kinds of water samples) (**Figure 4A** and **Table 3**). Among these antibiotic groups, *tet(44)*, *tet(M)*, *tet(W)*, *tet(Q)*, and *tet(O)* encoding resistance to tetracycline (**Figure 4B**), *aph(3)-III*, *aadA6*, and *aadA1* encoding resistance to aminoglycoside (**Figure 4C**), *mef(A)*, *mef(B)*, and *erm(F)* encoding resistance to macrolide (**Figure 4D**), *floR* and *optrA* encoding resistance to phenicol (**Figure 4E**) and three *sul* resistance genes (**Figure 4F**) were the most frequently identified genotypes.

**Table 3:** The number of co-occurrence of genotypes among the four kinds of water samples.

Antibiotics	DNA	Number	The same genotypes among the four kinds of water samples
Tetracycline	BAC	24	*tet(M) tet(W) tet(O) tet(44) tet(Q) tet(32) tet(C) tet(40) tet(X) tet(G) tet(S) tet(H) tet(A) tet(L) tetA(P) tet(T) tet(Y) tet(B) tet(Z) tetB(P) tet(39) tet(36) tet(33) otr(C) tet(D) tet(E)*
	PHA	13	*tet(M), tet(W), tet(O), tet(44), tet(Q), tet(32), tet(C), tet(X), tet(G), tet(S), tet(A), tet(L), tet(39)*
Macrolide	BAC	25	*mef(A) mef(B) erm(F) msr(D) lnu(C) lnu(B) ere(A) erm(B) erm(G) srm(B) erm(42) car(A) msr(E) lnu(F) ole(B) ole(C) tlr(C) mph(E) erm(Y) ere(B) erm(35) erm(A) erm(Q)erm(T) erm(35) mph(A)*
	PHA	9	*mef(B), erm(F), lnu(C), lnu(B), erm(B), srm(B), msr(E), lnu(F)*
Aminoglycoside	BAC	40	*aph(3′)-III, ant(6)-Ia, aadE, aac(6′)-aph(2″), ant(6)-Ib, aadA1, strA, aadA17, aadA8b, strB, aadA24, aadA5, aac(6′)-IIa, aac(6′)-Iai, aph(3′)-Ic, aph(4)-Ia, ant(3″)-Ih-aac(6′)-IId, aac(3)-Iva, aadA6, aadA8, aadA13, aph(2″)-Ic aph(2″)-Ie, aadA22, aadD, aph(2″)-Ib, spc, aac(6′)-Ia, aph(3′)-Ia, aadA11, aph(3′)-Ib, npmA, aac(3)-II, aac(6′), str, aac(6′)-Im, aadA16, aadB, aph(3′)-IIa*
	PHA	14	*aph(3′)-III, ant(6)-Ia, aadE, aac(6′)-aph(2″), ant(6)-Ib, aadA1, strA, aadA17, aadA8b, strB, aadA24, aac(6′)-IIa, aac(6′)-Iai, ant(3″)-Ih-aac(6′)-IId, aph(2″)-Ic*
Phenicol	BAC	15	*floR, cat, optrA, catQ, cmlA1, cmx, catB3, fexA, cml, catB8, catP, cfr, catB2, cat(pC194), catA2*
	PHA	5	*floR, optrA, catQ, catB3, fexA*
Sulphonamide	BAC	3	*sul1, sul2, sul3*
	PHA	3	*sul1, sul2, sul3*

With a careful analysis of these genes we found that all of these highly abundant ARGs have similar properties according to their mechanisms of action. Genes encoding major facilitator superfamily (MFS) [including *floR*, *mef(A)*, *mef(B)*, and *erm(F)*] (Coyne et al., 2011), genes encoding ATP-binding cassette (ABC) transporter family (including *optrA* gene that mediated antibiotic resistance through the ribosomal protection) (Sharkey et al., 2016; Wilson, 2016), and genes encoding ribosomal protection [including genes *tet(44)*, *tet(M)*, *tet(W)*, *tet(Q)*, and *tet(O)*] (Chopra and Roberts, 2001) were the most identified ARG groups. These results were similar to previous studies using raw sewage samples obtained from (i) Pittsburgh, Pennsylvania, United States, (ii) Barcelona, Spain; and (iii) Addis Ababa, Ethiopia (Cantalupo et al., 2011; Lekunberri et al., 2017a). Obviously, we clearly observed that their presence and distribution in phages and bacteria had geographic independence and the levels of these genes were also not fundamentally changed by wastewater treatment or pond natural degradation. Thus, whether the phages are more likely to carry these types of resistance genes in the contribution to antibiotic resistance in environment remains an open question.

On the other hand, according to a previous study that though phages rarely encode ARGs in the human-associated viromes, a relative high abundance of genes encoding ribosomal protection protein in tetracycline resistance genes group was detected in the human feces (Lekunberri et al., 2017a), which showed a similar trend to our results. Interestingly, in this study, we precisely identified these genes as the *tet(44)*, *tet(M)*, *tet(W)*, *tet(Q)*, and *tet(O)*. As for the highly abundant and gene groups simultaneously occurring between an animal-related environment and a human-related environment, our alarming findings may indicates that phages serve as a bridge to spread the ARGs from animals to human.

In addition, the β-lactamase, quinolone and trimethoprim resistance genes, *bla_OXA-347_*, *aac(6)-Ib-cr*, and *dfrA1*, were the most abundant in their own groups, even though their abundance was less than that of the top five antibiotic resistance groups, but they should be of particular concern to address future research (**Supplementary Figures S2–S4**). The gene *bla_OXA_* groups were the most identified sub-groups D belonging to the β-lactamases (Subirats et al., 2016). The *aac(6′)-Ib-cr* is a variant gene, similar to the widespread aminoglycoside N-acetyltransferase *aac(6′)-Ib* (Vetting et al., 2008). Gene *dfrA1* has previously been associated with classes 1 and 2 integrons and co-occurred with *aadA1* and *sul1* (van Essen-Zandbergen et al., 2009; Kehrenberg and Schwarz, 2011; Roth and Breaker, 2013). These findings suggest that popular genotypes and integrons may be an important connector for phages to spread antibiotic resistance and therefore may accelerate the evolution of these genes in the process of communication with bacteria.

### Assessing the Contribution of Phages to Antibiotic Resistance Genes

We next assessed the contribution of phages to transfer antibiotic resistance in swine feedlot wastewater and used three important indices: ARG abundance in bacteria, ARG abundance phage and their ratio values (RA values). The RA value was used as an estimator of “spreading ability.” In this study, we consider that the spreading ability depends on the abundance as well as the mutual exposure and exchange probabilities or communication frequencies between phage and bacteria. Therefore, a simple measure of ARG abundance does not necessarily correlate with a greater probability of ARG transfer by transduction.

**FIGURE 5:**
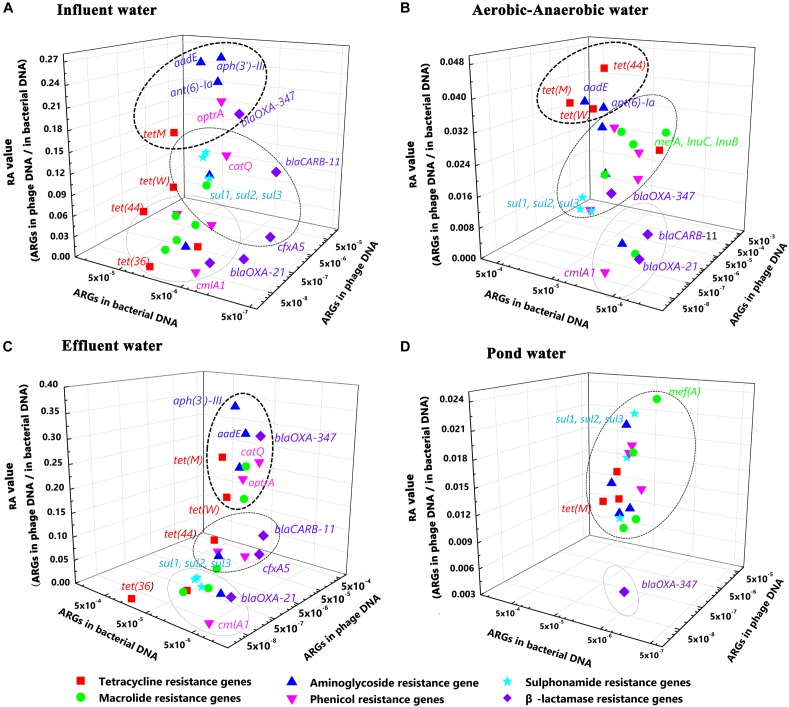
The five most abundant genotypes of the six most abundant antibiotic groups in three dimensional scatter plots. The *X*-axis represents the relative ARG abundance in bacterial DNA. The *Y*-axis represents relative ARG abundance in phage DNA. The *Z*-axis represents the Ratio, **(A)** Influent water; **(B)** Aerobic-Anaerobic water; **(C)** Effluent water; **(D)** Pond water.

For RA value measurements, we chose the five most abundant genotypes from the top six most abundant antibiotic resistance groups. The RA values were drawn as three dimensional scatter plots to better represent the transduction abilities of different ARG types (**Figure 5**). **Figure 5** shows that the ARGs were divided into three categories from the top to the bottom. However, the ARGs in wastewater treatment water samples showed a diversity feature for ARGs (**Figures 5A–C**) whereas they gather together in the pond water samples ARGs (**Figure 5D**). On the other hand, the SA values in pond water samples were lower than that in the wastewater treatment water samples. These results again indirectly demonstrate that animal feedlot wastewater treatment is an important ARG reservoir and is an important contributor to the spread of antibiotic resistance.

Among the three water samples from the wastewater treatment system, we found that the intermediate water and effluent water representing aerobic and facultative digestion procedures did not significantly alter ARG distribution and composition characteristics (**Figures 5A–C**). Meanwhile, the aminoglycoside resistance genes *aadE* and *aph(3′)-III*, the tetracycline [*tet(M)*] and phenicol (*optrA*) resistance genes always possessed the highest RA values. At the same time, these genes were also the most abundant in bacterial and phage DNA. Obviously, these genes should be of high priority in future studies.

Surprisingly, though the relative abundance of β-lactamase genes was lower compared with the other antibiotic groups, some of the β-lactamase ARGs still possessed relatively high RA values, indicating a potential risk especially for *bla_OXA-347_* that was detected with a very high RA value. On the other hand, considering combining the high abundance results of β-lactamase ARG in the animal-related environmental samples using qPCR method from other studies, future studies for assessing the contribution of phages to antibiotic resistance should also include screening for β-lactamase genes rather than only for *aadE*, *aph(3′)-III*, *tet(M)*, and *optrA*.

## Conclusion

We present the first study to use metagenomics to analyze the contribution of phages to antibiotic resistance in water samples from swine feedlot wastewater. Our data showed that aerobic and facultative digestions did not significantly decrease the abundance of ARGs in the phage DNA fractions. At the same time, ARGs from phage DNA could be an important reservoir of ARGs and play a major role in the spread of antibiotic resistance by transduction. Moreover, ARG distributions in phages and bacteria were independent of the geographical location and their trends were not fundamentally changed by wastewater treatment or natural degradation in pond water. Tetracycline resistance genes were always the most abundant ARG group and phages were more likely to harbor ABC transporter family and ribosomal protection genes. Surprisingly, the colistin resistance gene *mcr-1* was detected in our samples, which may imply that phages are another driving force for the horizontal genes transfer in the environment and have a potential risk to humans. Besides, further analysis of the spreading ability of different ARGs, *aadE*, *aph(3′)-III*, *tet(M)*, *optrA*, and *bla_OXA-347_* should be given a high priority in future research.

## Author Contributions

ZZ and YS designed the study and prepared the manuscript. MW, PL, XX, and JZ performed the experiments. WX analyzed the high-throughput sequencing data. All authors discussed the results. MW analyzed qPCR data and drafted the manuscript.

## Conflict of Interest Statement

The authors declare that the research was conducted in the absence of any commercial or financial relationships that could be construed as a potential conflict of interest.

## Footnotes

1 http://www.mg-rast.org/

2 http://www.mg-rast.org/linkin.cgi?project=mgp86199

## Abbreviations

ARB: antibiotic resistant bacteria
ARGs: antibiotic resistance genes
HGT: horizontal gene transfer
