# Supplementary material for: Metagenomic Insights Into the Contribution of Phages to Antibiotic Resistance in Water Samples Related to Swine Feedlot Wastewater Treatment
Source: Front Microbiol. 2018 Oct 16;9:2474. doi: 10.3389/fmicb.2018.02474 (PMC6232670; doi:10.3389/fmicb.2018.02474)
Supplement: Supplementary file 1 [file Data_Sheet_1.docx]

Supplemental Materials

Metagenomics insights into the contribution of phages to antibiotic resistance in water samples related to swine feedlot wastewater treatment

Mianzhi Wang^1,2^, Wenguang Xiong^1,2^, Peng Liu^1,2^, Xiying Xie^1,2^, Jiaxiong Zeng^1,2^, Yongxue Sun^1,2*^, Zhenling Zeng^1,2*^

^1^ National Risk Assessment Laboratory for Antimicrobial Resistance of Animal Original Bacteria, South China Agricultural University, Guangzhou, China

^2^ Guangdong Provincial Key Laboratory of Veterinary Pharmaceutics Development and Safety Evaluation, College of Veterinary Medicine, South China Agricultural University, Guangzhou, China

Abbreviations: ARB (antimicrobial resistant bacteria), ARGs (antimicrobial resistance genes), HGT (horizontal gene transfer)

* Corresponding author

Tel: +86-2085284313

E-mail address:

[sunyx@scau,edu.cn](mailto:sunyx@scau,edu.cn) (YX.Sun)

[zlzeng@scau.edu.cn](mailto:zlzeng@scau.edu.cn) (ZL.Zeng)

Pages: 4

Figure: 4

Tables: 1


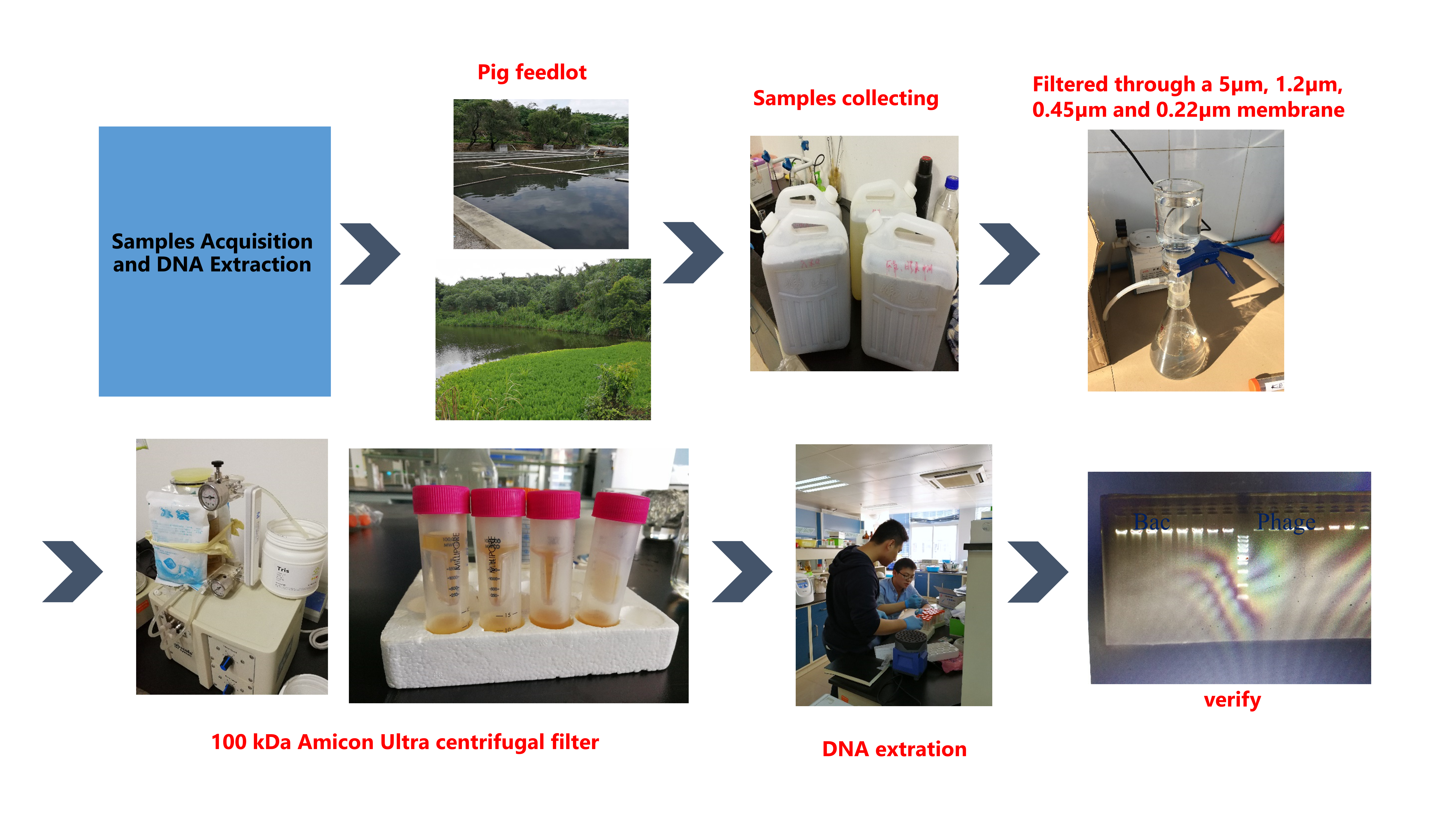


Fig S1: Schematic illustration of bacteriophage acquisition and DNA extraction.


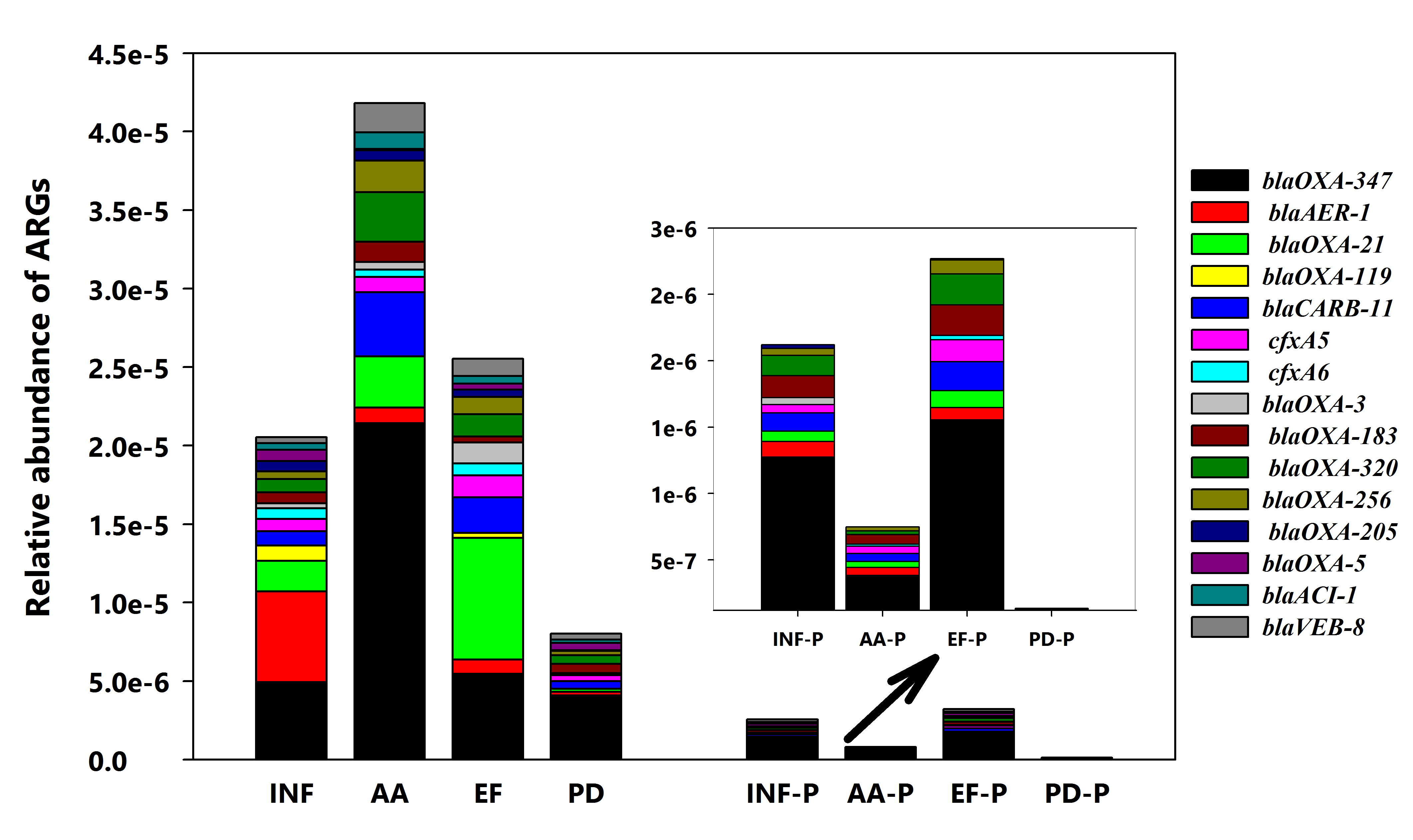


Fig S2：Distribution patterns of β-lactamases ARG types at different sampling locations.


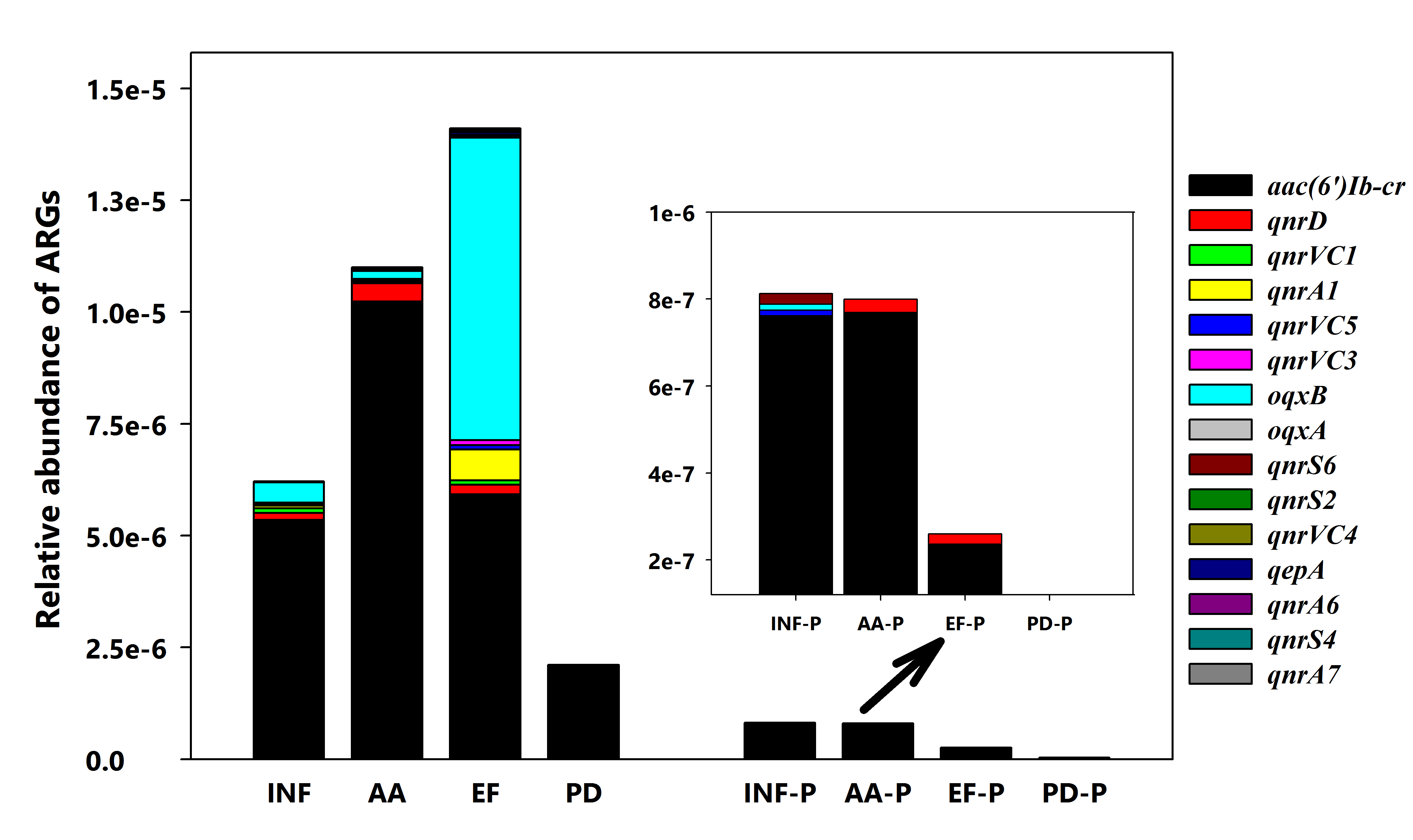


Fig S3：Distribution patterns of quinolone ARG types at different sampling locations


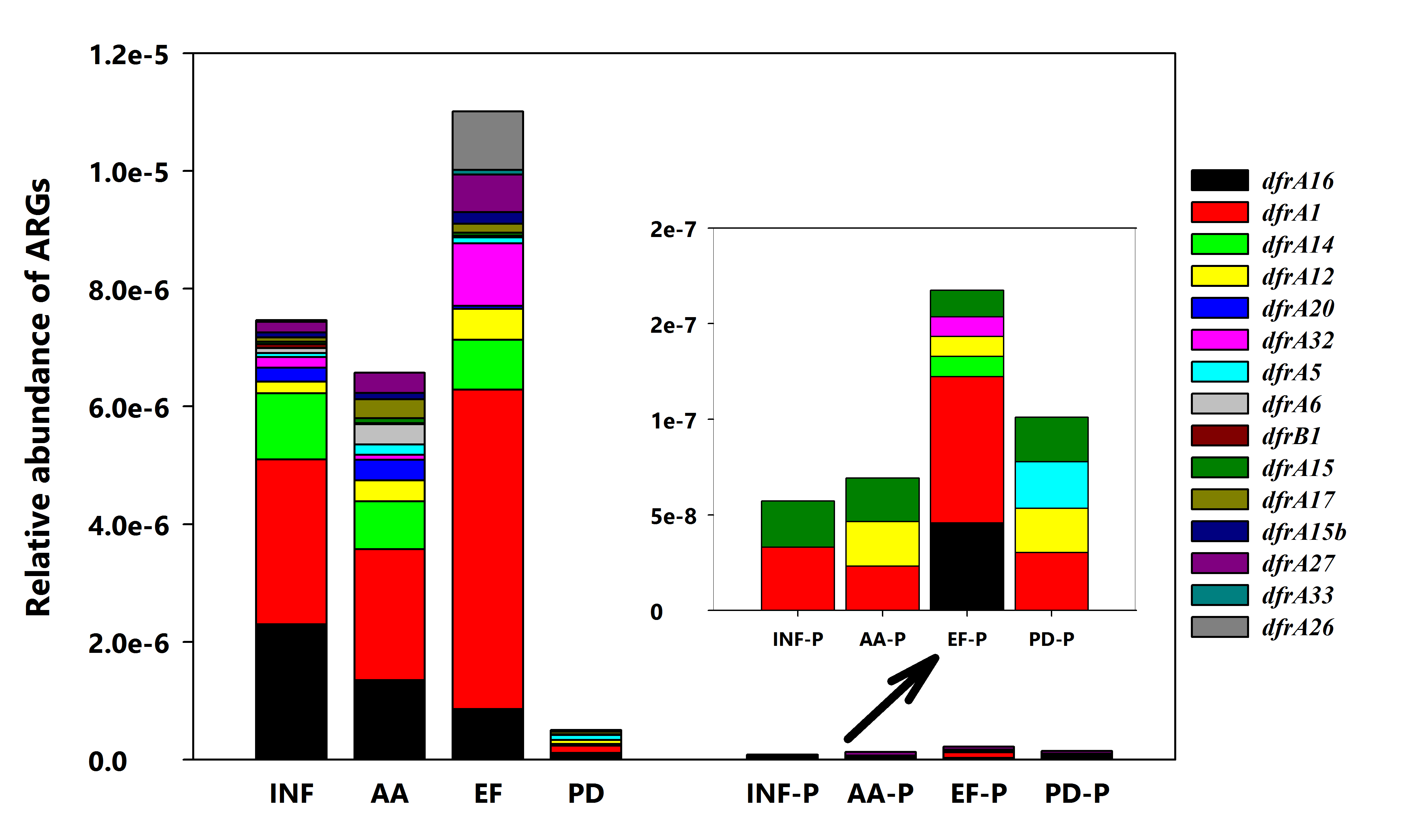


Fig S4：Distribution patterns of trimethoprim ARG types at different sampling locations

**Table S1**

Conditions for the detection of 16S rDNA (for conventional PCR).

| Groups | Genes | Primer sequence(5’-3’) | MT^b^ | bp | Reference |
| --- | --- | --- | --- | --- | --- |
| 16s rDNA | *16s-1* | F-GGTAGTCYAYGCMSTAAACG | Initial 95 °C denaturation for 5 min, followed by 35 cycles consisting of denaturation (95 °C for 30 s), annealing **(61.7 °C for 30 s)**, extension (72 °C for 45 s); Final extension step (72 °C for10 min) | 263 | (Koike et al., 2010) |
|  | *16s-2* | R-GACARCCATGCASCACCTG |  |  |  |
|  | *28f* | F-AAGAGTTTGATCCTGGCTCAGA | Initial 95 °C denaturation for 5 min, followed by 35 cycles consisting of denaturation (95 °C for 30 s), annealing **(61.7 °C for 30 s)**, extension (72 °C for 45 s); Final extension step (72 °C for10 min) | 1503 | Colomer-Lluch et al., 2014 |
|  | *1492r* | R-TACGGCTACCTTGTTACGACTT |  |  |  |

**Table S2**

Accession number and detailed information of 12 raw data in MG-RAST

| Sample name | Duplication | Accession number |
| --- | --- | --- |
| INF-B | B11 | mgm4801790.3 |
|  | B12 | mgm4801786.3 |
| EF-B | B21 | mgm4801792.3 |
|  | B22 | mgm4801784.3 |
| AA-B | B31 | mgm4801783.3 |
|  | B32 | mgm4801794.3 |
| PD-B | B41 | mgm4801791.3 |
|  | B42 | mgm4801793.3 |
| INF-P | P11 | mgm4801795.3 |
|  | P12 | mgm4801789.3 |
| EF-P | P21 | mgm4801787.3 |
|  | P22 | mgm4801797.3 |
| AA-P | P31 | mgm4801788.3 |
|  | P32 | mgm4801785.3 |
| PD-P | P41 | mgm4801798.3 |
|  | P42 | mgm4801796.3 |
